# Habituation of Fear—Israeli-Jewish Population during Protracted Belligerence

**DOI:** 10.3390/ijerph192316067

**Published:** 2022-11-30

**Authors:** Meytal Eran-Jona, Roni Tiargan-Orr, Stephen Z. Levine, Yehiel Limor, Mordechai Schenhav, Uzi Ben-Shalom

**Affiliations:** 1 Feinberg Graduate School, Weizman Institute of Science, Rehovot 7610001, Israel; 2 Department of Behavioral Sciences (MAMDA), Israel Defence Forces (IDF), Tel Aviv, Israel; 3 School of Public Health, University of Haifa, Haifa 3498838, Israel; 4 School of Communication, Bar Ilan University, Ramat Gan 5290002, Israel; 5 Hebrew and Jewish Studies Department, University of Strasbourg, CS 90032 Strasbourg, France; 6 Department of Sociology and Anthropology, Ariel University, Ariel 40700, Israel

**Keywords:** fears, low intensity conflict, public opinion, Israel, resiliency, COR theory

## Abstract

The identification of demographic factors of vulnerability and resilience in communities facing belligerent conflicts is increasingly relevant today. This representative study aims to examine the effect of protracted violence on the level of fear of the overall Israeli-Jewish population, and the role of the conflict on the connection between socio-economic factors and fears. Sixty-six representative samples were identified and surveyed from 2001 to 2019 (*n* = 37,190) that occurred during (*n* = 14,362) and between (*n* = 22,828) seven conflicts and non-conflict periods. Results show that during military conflicts, civilians declared less fears of physical injury comparing routine time; a slow trend of decline in the level of fears over time was observed; during routine periods, fear was associated with female-gender and with the lowest income level group. Ultra-orthodox and Religious respondents had significantly less fear than the secular and traditional respondents. During military conflicts, the results changed significantly, mainly for the lowest income group, women and ultra-orthodox.

## 1. Introduction

### 1.1. Settings

Between the beginning of the new millennium and the time this study was conducted, Israel has faced ongoing terror attacks targeted to harm the civilian population and with a series of Low Intensity Conflicts (LICs) within its borders. A Low Intensity Conflict is defined as a political-military confrontation between contending states or groups below conventional war and above the routine, peaceful competition among states. LICs range from subversion to the use of the armed forces [1]. These conflicts involve many “third-side actors” such as NGOs, the global mass media and local citizens [2,3,4,5]. The current study covers the 18-year period from 2002 to 2019 among the Jewish population of Israel. The beginning of the period can be marked in September 2000, with the beginning of the so-called “Second Intifada” (or in Israeli terminology: “wave of terrorist attacks”). The period described is considered to be stormy from a public security point of perspective and contained incidents of terror attacks against civilians and soldiers all around Israel.

During the study period seven peak events occured. These events framed in the Israeli media as LICs, directly affected, in diverse ways, a large part of the country’s residents or even all of them. During these events the Israeli defense forces (IDF) was activated (or there was an understanding that it was about to be activated, as happened during the Second Gulf War). Each of these events was characterized by some or all of the following: defining a state of emergency at one level or another; operating emergency regulations throughout the country or part of it; formal naming of the event; recruitment of reserve army forces in large numbers; a wide media coverage; at the time of these events, there was almost no presence for media reports unrelated to the fighting. Six of these events are defined militarily as a LIC: operation “Defensive Shield” (2002), “the Second Gulf War” (2003), operation “Summer Rains” (2006), operation “Cast Lead” (2008-9), operation “Pillar of Defense” (2011) and operation “Protective Edge” (2014). The seventh event, “the Second Lebanon War” (2006) was the most intense and the only military conflict during our study that was formally defined as “war” by the Israeli government, although it can be considered as a LIC event as well [6].

When considering Israel it is noteworthy that Israel is a geographically small country with a diverse demographic composition whose entire population lives under constant threat of terror and war. The issues of threat and war are regularly discussed in the Israeli public debate almost daily, influencing people’s perceptions and feelings. Moreover, the war on terror is in close proximity to people’s homes and neighborhoods [7,8,9,10,11]. Studies estimate that approximately half of the Israelis have been directly exposed or have friends or family who were exposed to terrorist attacks [12,13,14]. Therefore, the Israeli context of terror has unique features compared to other Western countries, where terror attacks during the last years were less frequent, and wars occurred further from home. However, relatively little is known about fear in the general population.

### 1.2. Military Conflicts and Their Psychological Implications

During recent decades, significant changes occurred in the characteristics of war. The change is multidimensional and is influenced by global, cultural, social, and technological aspects [2,3,5,6]. One of the main effects of the changes is the increase in the frequency of low-intensity conflicts. The psychological impact of war, terror, and LICs on civilians and societies has been widely studied, dealing with the vulnerability and resiliency of communities and individuals who facing terrorism and war [15,16,17,18,19,20,21,22,23,24,25,26,27]. Researchers conducted in many countries that indicate that armed conflicts and terror attacks have a significant impact on emotions, fears, stress, and posttraumatic stress disorder [7,12,13,19,20,27,28,29,30,31,32,33,34,35,36].

These questions are increasingly relevant today, as terrorism spreads and communities worldwide are threatened by terror attacks [27]. Therefore, it is crucial to learn about the factors that contribute to psychological distress, fears, and resilience in front of the terrorist threat and turbulent environments.

### 1.3. Fears during Armed Conflict and the COR Theory

Bar-Tal [28] define the emotion of fear as a specific subjective aversive feeling that arise when one perceives a threat or danger to oneself and/or one’s society and enables an adaptive response. Existential fear was found as one of four sources of stress effecting Israeli families during wartime (the other sources of stress were ambiguity, war-related hardships, and inter-family strain [10].

Hobfoll Conservation of Resources theory (COR) has been adopted to understand how people are affected by terrorist attacks, war, or disaster [31,37,38,39]. The COR theory [40,41] is based on the supposition that people strive to retain, protect and build resources and that what is threatening to them is the potential or actual loss of these resources. Wartime creates the context wherein there is the possibility for damage to people’s valuable resources, their homes and property, their income and livelihood, their psychological well-being, and even their lives.

Building on Hobfoll COR theory, our assumption is that fear is formed at the intersection between the individual (strength, resources) and the context (the social-political and the security state). Therefore, fears like stress are primarily a consequence of threat of loss or actual loss of resources, both material and psychosocial.

### 1.4. Vulnerability and Demographic Variables

The COR theory provides a unified explanation for the reasons that demographic variables may be related to vulnerability. Low education, ethnic minority status, lower age, female genderand low income have been previously found to be associated with risk of PTSD and stress following terrorism [12,13,17,22,26,31,42,43,44]. It is generally thought that these demographic indicators reflect having limited access to psychological and financial resources [17,31]. Gender is a variable that has been identified in many studies as differentiating women from men in their responses to threats regarding the levels of fear, stress, PTSD and resiliency [13,17,44,45,46,47,48]. Age was found to be related to the ability to handle different kinds of crises and disasters, most notably in the extremes age groups, with children on the one hand and the elderly on the other hand [42,46,49].

Researchers have suggested that the power of religion may help people cope with grievances and extreme situations such as disasters [50]. In a study conducted in Israel during the Second Intifada, religious Jews reported the fewest and least severe symptoms of stress-related complaints, the least sense of personal threat, and the highest level of functioning [32]. They were also found to be more resilient and with less tendency to develop chronic distress [31]. No literature that provides any evidence suggesting that there is an effect of parenthood on psychological vulnerability during military conflicts was identified.

Despite its limitations, the study has three advantages. First, it is the first long-term study that addresses the issue of fear within the Israeli-Jewish population spanning a period of eighteen years (2001–2019). Second, the study design is quasi-experimental, which uses a before, during, and after seven military conflicts study design. Thus, it is the first long-term study that compares emotions of fear in the Israeli context during periods of war and LICs and compares it with fears during periods of relative quiet. Third, the sample is representative, and the sample size is larger than other studies undertaken in this field.

### 1.5. Research Questions and Hypothesis

Consequently, this article focuses on three research questions:

1. What is the effect of LIC on the fear level of Israeli-Jews during the conflict?

We hypothesized that during LICs, the level of fear would increase compared to routine periods.

2. Can we identify a change in fear level trend while LIC events accumulate?

We assumed that with the accumulation of LICs, a process of habituation will take place, and the level of declared fears of the impact of LICs will decrease.

3. Which Demographic and socio-economic characteristics are associated with high fear levels during LIC?

Building on the COR theory, we assume that Israeli citizens with low education, young adults, female sex and low income will be more vulnerable to fear during LICs compared to periods unexposed to LIC.

## 2. Methods

### 2.1. Population, Data Collection, and Sample

Cellular and landline telephone numbers were randomly selected to obtain nationally representative samples of Israeli-Jewish adults aged 18 and over who live in Jewish or mixed Jewish-Arab cities and municipalities, such as Haifa, Jaffa or Acre. Data was collected between March 2001 and May 2019 in 67 representative surveys of the Israeli-Jewish population.

The sample was determined independently for each of the 67 points in data collection. Each of the samples represented the distribution in the Israeli-Jewish population. The overall response rate among eligible responders was about 25%. Interviews were conducted in Hebrew by trained interviewers. Data was gathered during periods of routine (between military conflicts) and during seven military conflicts, as mentioned above.

The overall data consisted of 37,190 participants, 22,871 interviews conducted during routine periods and 14,363 interviews conducted during LIC periods, as seen in Table 1.

### 2.2. Measures

The surveys focused on three main issues: how the Israeli public perceives the security situation, public endurance and resilience; and the degree of trust in the IDF.

The current paper is based mainly on two parameters that examined fears. The first is more general and may be relevant in everyday situations (“I am afraid of the future”). This parameter was examined in all surveys. The second parameter is relevant mainly during security tension (“I am afraid of being physically injured due to the current security situation”). Participants responded on a Likert scale ranging from 1 (strongly disagree) to 5 (strongly agree). In order to characterize respondents who expressed strong fear of the future or strong fear of physically injury, the variables were dichotomized into high level (“strongly agree” and “agree”) or low level of fear (all other responses).

An additional measure, the “Fearful group”, was created: it includes respondents who “strongly agreed” with both questions (whenever they were asked). This group was designed in order to distinguish people who tend to worry more than others.

### 2.3. Analytic Plan

The analysis consisted of descriptive statistics; logistic regression models; and logistic regression stratified by periods: during the routine time, during all seven LICs and separately during the second Lebanon War, which was considered the most intense LIC event. The phrasing of the questions, the nature of the sampling and the manner of questioning remained consistent over the years, although not all the questions were asked in all surveys.

The demographic covariates were: sex, religious faith (ultra-orthodox, religious, traditional, secular), age groups (18–24, 25–34, 35–44, 45–54, 55–64, 65+), parenthood of young children (parents who are under the age of 50, assuming that this group applied to the majority of parents of minor children), Educational level and declared income level.

## 3. Results

### 3.1. The Effect of LIC on Fear Level of Israeli Jews during Conflict

In order to examine the first research question, we will first observe the distribution of the respondents who replied positively to both fear questions and the frequency of the “fearful group”—during LIC periods, during the second Lebanon war, and routine periods (presented in Table 2).

Table 2 indicates that for the question that examined fears of the future, there was no significant gap between routine and LICs periods (47%, 48%). A slightly higher percentage of respondents declared fear during the Second Lebanon war (51%) compared to other military conflicts 47%.

During routine periods, a significantly (*p* < 0.05) higher rate of Israeli citizens expressed concern about getting physically injured as a result of the security situation, compared to periods of military conflicts (30%, 23%). No significant difference was found by comparison of LICs and Second Lebanon War (24%).

3.2% of the respondents belong to the fearful group during routine periods and 4.9% during military conflicts (*p* < 0.05).

Statistically significant Spearman’s r correlations were observed between the two fear questions during routine periods and during military conflicts (r = 0.434, *p* < 0.000; r = 0.402, *p* < 0.001; respectively).

### 3.2. Changes and Trends in Fear Level along the Entire Period

**Figure 1:**
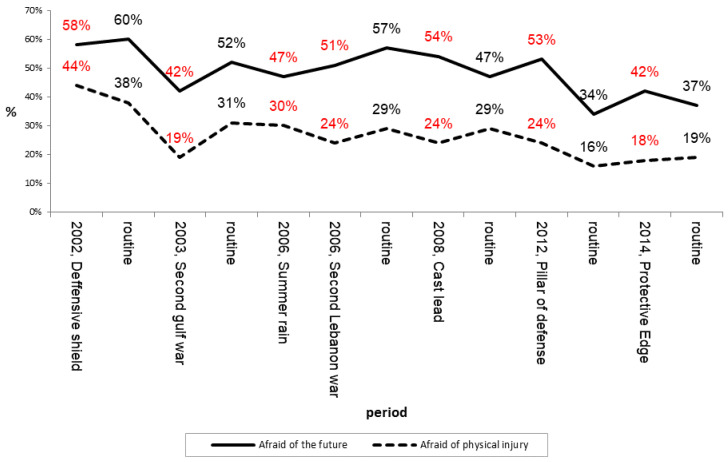
Percentage of respondents who answered positively during routine periods and LIC periods, 2002–2019. Red percetage present surveys collected during Lic periods, Black percetage present surveys collected during routine.

In order to examine the second research question by trying to identify a trend of change in the public’s fear level while LIC events accumulating, we examined (Figure 1) the percentage of respondents who replied positively (“strongly agree” or “agree”) to both statements across time.

As shown in Figure 1, it appears that during most of the LICs periods, the level of fear of being physically injured is generally (surprisingly) lower compared to the adjacent routine periods, except for one event, “Defensive Shield” operation in 2002.

As for “fear of the future”, the results are fluctuating, both during routine and during LICs. This finding may indicate that the existence of a military conflict is not enough to explain changes in the fear level.

Another interesting finding is that it can be claimed with caution that as time goes on and LIC events are accumulating—the fear level decreases, for both parameters, both comparing LICs and comparing routine periods.

### 3.3. Demographic and Socio-Economic Characteristics and LIC

In order to examine the last research question, multivariate binary logistic models were calculated to examine the likelihood of reporting fears on the three measures, regarding different socio-demographic variables.

The first model examined the association between socio-demographic factors and the fearful index. Results presented in Table 3 demonstrate that women compared to men (OR = 2.08, CI = 1.80, 2.41; *p* < 0.01) and the lowest income level group (OR = 1.54, CI = 1.27, 1.86; *p* < 0.01) compared to the other income level groups displayed a significantly higher likelihood of fear. In contrast, the ultra-orthodox group (OR = 0.64, CI = 0.45, 0.94; *p* < 0.01) and religious group (OR = 0.70, CI = 0.54, 0.90; *p* < 0.01) displayed a significantly reduced likelihood of fear compared to traditional and secular Jews.

The second model examined the connection between socio-demographic factors and fear of the future. Women compared to men displayed a significantly higher likelihood of fear of the future (OR = 2.22, CI = 2.10, 2.35; *p* < 0.05). The ultra-orthodox group (OR = 0.67, CI = 0.59, 0.77; *p* < 0.01) and religious group (OR = 0.81, CI = 0.74, 0.89; *p* < 0.01) displayed a significantly reduced likelihood of fear of the future compared to traditional and secular Jews.

The third model examined the connection between demographic factors and fear of physical injury. Results presented in Table 3 showed that for this measure also, women compared to men (OR = 1.71, CI = 1.58, 1.85; *p* < 0.01) were significantly at higher risk of fear of physical injury.

The lowest income level group compared to the other income level groups (OR = 1.44, CI = 1.28, 1.61; *p* < 0.01), younger age (18–34) compared to the elderly (OR = 1.45), were also at higher risk of fear of physical injury. The ultra-orthodox group (OR = 0.70) displayed a significantly reduced likelihood of fear.

### 3.4. Demographic and Socio-Economic Characteristics during and between Armed Conflicts

In order to complete the examination of the third research question, a stratified by periods logistic regression models was conducted: during routine periods, during LICs and separately during the second Lebanon War, for all the measures.

#### 3.4.1. The “Fearful” Group

Comparing routine periods and LIC periods (Table 4), the most prominent changes related to Gender and Socio-Economical Status (SES, which are expressed by Income level or Educational level):

As for Gender, during routine times, the likelihood of belonging to the fearful group among women is high (OR = 1.84, CI = 1.48, 2.29; *p* < 0.01), and during LICs, it is even higher (OR = 2.28).

As for SES, during LIC periods the likelihood of belonging to the fearful group among the lowest Non-Academic respondents (OR = 3.20, *p* < 0.01) is higher compared to routine periods (OR = 0.83, *p* = 0.13). We can also observe that during the Second Lebanon War, the likelihood of belonging to the fearful group among the lowest income level (OR = 3.57, *p* < 0.01) is higher compared to routine periods (OR = 1.56, *p* < 0.01).

#### 3.4.2. Fear of Physical Injury

Table 5 presents a binary logistic regression model for the parameter “I am afraid of being physically injured as a result of the current situation”, during routine and during LICs. Results show that during the routine, fears are strongly connected with women (OR = 1.64, CI = 1.46, 1.84; *p* < 0.01) compared to men; to the lowest income group compared to all other groups (OR = 1.30, CI = 1.09, 1.55; *p* < 0.01) and significantly reduced likelihood to Ultra-Orthodox Jews (OR = 0.61, CI = 0.44, 0.85; *p* < 0.01).

No significant differences compared to LICs model, except for age: while no difference was found during the routine, for younger age groups, especially 25–34, during LICs the likelihood of fear of being physically injured is higher compared to routine periods (OR = 1.66 during LICs compared to OR = 1.01 during routine periods).

#### 3.4.3. Fear of the Future

Table 6 demonstrates a stratified analysis of binary logistic regression model for the parameter “I am afraid of the future”. For this parameter, distinctions between different population groups are much more evident during LICs than during routine periods, compared to the other two measures.

Examining gender, the likelihood of women to report fear of the future during routine periods is high compared to men (OR = 1.83, CI = 1.68, 1.99; *p* < 0.01). During LIC events in general, and especially during the Second Lebanon War the likelihood is significantly higher compared to routine periods (OR = 2.61, CI = 2.42, 2.81; *p* < 0.05 during LIC periods, OR = 2.96, CI = 2.60, 3.34; *p* < 0.05 during the Second Lebanon War).

Regarding age: while no significant difference was found during routine periods—the likelihood of fear of the future for age groups 35–44 during LICs is higher (OR = 1.31 during LICs, OR = 1.52 during the Second Lebanon War).

Regarding Income level, during routine, no significant correlation was found, while during LICs the lower income group show greater concern about the future compared to the other income groups (OR = 0.1.3 during LIC periods and OR = 1.4 during Second Lebanon War).

## 4. Summary and Discussion

The current study aims to contribute to the growing literature in the field of military conflicts and fear by addressing the questions of vulnerability and resiliency of people and communities in the face of Terror attacks, Military Confrontations or LICs based on a nationally representative sample of Israelis Jewish population spanning over 18 years.

Our first research question tried to address the effect of LIC on fear level of Israeli Jews during the conflict. Comparing fear levels during LICs to routine periods, the study shows somewhat surprising evidence of decreased fears of getting physically hurt in the Israeli-Jewish population during LICs, compared with intervals without military conflict.

Our second research question aimed to reveal trends of change in fear level while LIC events are accumulating. We can say with caution that as time goes and the experience of the Israeli public in coping LIC events increase—the level of fear decreases, both comparing LIC events and comparing routine periods. After two decades of ongoing military conflicts inside and along Israel’s borders, it seems that armed conflicts have made most Israelis resilient in the face of threat.

Our third question tried to find demographic and socio-economic characteristics that are associated with fear level during LIC, as a test of Hobfoll’s COR theory (1989). The results offer evidence of Socio-Economic, faithand demographic factors that link to fears, which is consistent with Hobfoll’s COR theory.

We would like to offer a tentative discussion of the current study results.

First, the study illustrates the uniqueness of the Israeli context during the study’s period, in which Israeli citizens were coping with ongoing military conflicts and terror attacks [51]. A military conflict was found to be an event that enhances solidarity and social cohesion of the Israeli Jewish citizens and increases the trust in the military and its ability to tackle the threat [6]. Thus, fighting Hamas in the south of Israel, or Hezbollah on Israel’s northern border creates a feeling that the threat is addressed and raises hope for a quieter and safer reality in the future. These findings may suggest that people attain resilience in part due to the military actions and in part due to a process of adaptation.

We assume that the current findings differ from other research that is focused on reactive psychopathology (mainly PTSD), unlike this study which examined “normal” fear, and not severe or existential fear. Moreover, the questions asked might be understood somewhat differently when measured during a LIC and when measured during times of relative calm: In times of military confrontation, respondents may have a clearer understanding of the security situation and, therefore can perhaps make a sounder assessment of their probability of being hurt. However, when these items are being assessed while there is no tangible threat, the assessment is much more abstract and frightening. In that case we can assume that military conflicts that were studied for this paper were assessed by the Jewish-Israeli citizens as less frightening than expected.

Our findings suggest that individuals and collectives can, as Jarymowicz and Bar-Tal [52] argue, establish an orientation of hope, which allows change in situations dominated by fear. A similar explanation may be that, as Pliskin et al. [53] suggested, when there is an armed confrontation, individuals believe that the proper response is: “we should be resilient if we don’t want the enemy to win”. Therefore, the findings can also be another expression for the “rally around the flag effect” [54,55].

The study results suggest that the decrease in the level of fears during LICs is not uniform for all research groups. This is consistent with Hobfoll’s COR theory that postulates that groups with fewer coping resources are more likely to be influenced by a stressful external event and more vulnerable. Indeed, the following were associated with a greater likelihood of fear: female gender and low Socio Economic status. On the other hand, religiosity was found as a source of resilience, as other have reported [31].

The results pertaining to gender are consistent with the literature on fears, stress and PTSD [56], as are the findings on lower SES. Comparison between logistic regression models explaining fears during periods of routine and between LICs shows that the most prominent changes were related to SES.

Though we believe that our study is innovative in some aspects, it has several limitations. First, the main limitation is that Israeli Arabs were not included in the study sample. Therefore, the current study only represents the Israeli-Jewish population who are the majority ethnic group (about 80% of the entire Israeli population). Second, we did not examine reactive psychopathology, such as PTSD. Third, despite the importance of voting patterns and political positions in understanding reactions to military conflicts, we, unfortunately, do not have such information. Thus, further research is warranted to broaden the research population to enhance the generalizability of the current findings and to enhance the psychological aspects of reactions to ongoing military and political conflicts in civilian populations.

## Figures and Tables

**Table 1:** List of surveys by dates, number of participations and LIC context.

Date	*n*	Military Context
April 2002	1326	Operation “Defensive Shield”
May 2002, August 2002, February 2003	1481	Routine
March 2003	1232	Second Gulf War
August 2003, January 2004, May 2004, June 2004, September 2004, January 2005, May 2005	2823	Routine
July–August 2005 (Before, during and after the “disengagement” from the Gaza Strip)	2371	Routine
January 2006	591	Routine
June 2006	1061	Operation “Summer Rains”
July–August 2006	4369	Second Lebanon War
August (end) 2006, January 2007, May 2007, August 2007, August 2008	2265	Routine
December 2008–January 2009	1220	Operation “Cast Lead”
December 2009, October 2010, October 2011	2666	Routine
November 2011	873	Operation “Pillar of defense”
May 2013	1235	Routine
July–August 2014	4281	Operation “Protective Edge”
October 2015–May 2019, 14 surveys conducted during this period	9396	Routine
Total participants during Routine	22,828	
Total participants during LIC	14,362	
Total participants in all surveys	37,190	

Note: A number of surveys (3–8) were conducted in each LIC, some on a daily or bi-daily basis.

**Table 2:** Percentage of respondents who answered positively to the statements during military conflicts, and routine periods.

	During Routine			During Military Conflict			During the Second Lebanon War		
	%	*n*	95% CI	%	*n*	95% CI	%	*n*	95% CI
“I am afraid of the future” (% responded “strongly agree” or “agree”)	47%	19,223	47%, 48%	48%	14,107	48%, 49%	51%	4108	49%, 52%
“I am afraid of being physically injured as a result of the security situation” (% responded “strongly agree” or “agree”)	30%	9370	29%, 31%	23%	11,046	22%, 24%	24%	2294	23%, 26%
Fearful group (% responded “strongly agree” for both questions)	3.2%	20,826	2.9%, 3.5%	4.9%	13,204	4.5%, 5.3%	3.5%	3775	3.0%, 4.0%

Note: Abbreviation: CI, 95% Confidence Intervals.

**Table 3:** Binary logistic model of the three fear measures.

Covariate	Levels of Covariate	Fearful Index, All (N = 18,595)				Afraid of the Future, All (N = 20,307)				Afraid of Physical Injury, All (N = 14,008)			
		*n*	*p*	OR	95% CI	*n*	*p*	OR	95% CI	*n*	*p*	OR	95% CI
Gender	Female	11,233	0.000	2.079	(1.797,2.406)	12,490	0.000	2.217	(2.095, 2.346)	8681	0.000	1.709	(1.581, 1.848)
Age	Age		0.068				0.001				0.000		
	18–24	2418	0.162	1.201	(0.929, 1.552)	2619	0.320	1.051	(0.953, 1.158)	1697	0.000	1.463	(1.274, 1.681)
	25–34	2841	0.009	1.415	(1.090, 1.837)	3065	0.007	1.152	(1.039, 1.276)	2223	0.000	1.446	(1.250, 1.671)
	35–44	3829	0.088	1.271	(0.965, 1.674)	4239	0.000	1.270	(1.140, 1.415)	2767	0.000	1.357	(1.156, 1.594)
	45–54	3664	0.015	1.368	(1.061, 1.762)	3995	0.002	1.176	(1.064, 1.301)	2868	0.000	1.324	(1.150, 1.524)
	55–64	2860	0.009	1.423	(1.094, 1.851)	3115	0.135	1.084	(0.975, 1.205)	2327	0.019	1.192	(1.029, 1.382)
Parenthood	Parents	5837	0.015	1.263	(1.047, 1.523)	6297	0.196	1.053	(0.974, 1.139)	4704	0.030	1.129	(1.012, 1.261)
faith			0.000				0.000				0.000		
	Ultra-orthodox	940	0.011	0.641	(0.454, 0.904)	1017	0.000	0.673	(0.589, 0.769)	661	0.000	0.697	(0.576, 0.844)
	Religious	2114	0.005	0.696	(0.539, 0.898)	2275	0.000	0.811	(0.740, 0.889)	1461	0.026	0.860	(0.754, 0.982)
	Traditional	4813	0.000	1.386	(1.190, 1.614)	5343	0.000	1.135	(1.061, 0.851)	3722	0.000	1.294	(1.184, 1.414)
Education			0.000				0.000				0.000		
	Non Academic	8746	0.212	1.124	(0.935, 1.351)	9674	0.000	0.831	(0.769, 0.898)	6444	0.000	0.743	(0.673, 0.820)
	Academic	6471	0.013	0.768	(0.624, 0.945)	6986	0.000	0.783	(0.721, 0.851)	4837	0.000	0.585	(0.525, 0.652)
Income			0.000				0.002				0.000		
	Low	3642	0.000	1.535	(1.265, 1.861)	4045	0.002	1.142	(1.049, 1.244)	2644	0.000	1.436	(1.284, 1.607)
	Medium-High	8308	0.628	0.957	(0.802, 1.143)	9039	0.976	1.001	(0.933, 1.071)	6218	0.612	1.025	(0.933, 1.126)
	High	1885	0.664	0.940	(0.710, 1.244)	2091	0.447	0.960	(0.864, 1.067)	1332	0.002	0.775	(0.661, 0.908)
constant			0.000	0.021			0.000	0.670			0.000	0.243	

Note: Abbreviations: *p*, *p*-value; OR, Odds Ratio; CI, 95% Confidence Intervals; SES, Socioeconomic status.

**Table 4:** Binary logistic model predicting the belonging to “Fearful” group during LICs and during routine, stratified analysis.

Covariate	Levels of Covariate	During Routine (N = 7566)				During LICs (N = 11,029)				During the Second Lebanon War (N = 3449)			
		N	*p*	OR	95% CI	*n*	*p*	OR	95% CI	*n*	*p*	OR	95% CI
Gender	Female	4373	0.000	1.838	(1.476, 2.288)	6860	0.000	2.278	(1.89, 2.775)	1441	0.000	2.134	(1.453, 3.133)
Age	Age		0.061				0.225				0.275		
	18–24	1216	0.057	1.467	(0.988, 2.177)	1202	0.900	0.978	(0.688, 1.390)	424	0.702	1.152	(0.558, 2.376)
	25–34	1256	0.079	1.454	(0.958, 2.207)	1585	0.061	1.383	(0.985, 1.943)	567	0.266	1.503	(0.733, 3.081)
	35–44	1658	0.733	1.080	(0.693, 1.685)	2171	0.032	1.484	(1.036, 2.128)	675	0.051	2.104	(0.996, 4.445)
	45–54	1385	0.064	1.483	(0.978, 2.248)	2279	0.108	1.300	(0.944, 1.791)	730	0.120	1.719	(0.868, 3.404)
	55–64	909	0.013	1.753	(1.127, 2.728)	1951	0.252	1.211	(0.872, 1.682)	610	0.896	0.949	(0.435, 2.069)
Parent-hood	Parents	2260	0.028	1.373	(1.034, 1.823)	3577	0.530	1.086	(0.840, 1.404)	1151	0.419	0.816	(0.498, 2.180)
faith Education			0.000				0.001				0.382		
	Ultra-orthodox	360	0.020	0.471	(0.250, 0.888)	580	0.243	0.780	(0.514, 1.183)	164	0.954	1.022	(0.479, 2.180)
	Religious	922	0.005	0.547	(0.358, 0.836)	1192	0.280	0.838	(0.607, 1.155)	383	0.093	0.532	(0.255, 1.112)
	Traditional	1899	0.005	1.400	(1.108, 1.768)	2914	0.002	1.383	(1.131, 1.692)	878	0.931	1.019	(0.669, 1.552)
			0.001				0.000				0.011		
	Non Academic	2918	0.125	0.828	(0.650, 1.054)	5828	0.000	3.202	(1.943, 5.279)	2035	0.829	1.142	(0.342, 3.817)
Income	Academic	2181	0.000	0.565	(0.421, 0.760)	4290	0.003	2.205	(1.318, 3.689)	1291	0.371	0.564	(0.161, 1.979)
			0.004				0.000				0.000		
	Low	1474	0.005	1.557	(1.144, 2.119)	2168	0.001	1.533	(1.195, 1.966)	661	0.000	3.574	(1.998, 6.394)
	Medium-High	3512	0.814	0.968	(0.736, 1.273)	4796	0.651	0.948	(0.751, 1.196)	1537	0.041	1.808	(1.024, 3.194)
	High	729	0.350	1.219	(0.805, 1.845)	1156	0.247	0.798	(0.544, 1.170)	371	0.858	1.090	(0.425, 2.798)
constant			0.000	0.026			0.000	0.008			0.000	0.011	

Note: Abbreviations: *p*, *p*-value; OR, Odds Ratio; CI, 95% Confidence Intervals; SES, Socioeconomic status. “During LICs” Includes the Second Lebanon War.

**Table 5:** Binary logistic model predicting “fear of getting physically injured” during LIC and during routine, stratified analysis.

Covariate	Levels of Covariate	During Routine (N = 5119)				During LICs (N = 8889)				During the Second Lebanon War (N = 2201)			
		*n*	*p*	OR	95% CI	*n*	*p*	OR	95% CI	*n*	*p*	OR	95% CI
Gender	Female	3056	0.000	1.640	(1.458, 1.844)	5625	0.000	1.812	(1.631, 2.014)	1308	0.000	1.760	(1.437, 2.156)
Age	Age		0.365				0.000				0.076		
	18–24	782	0.059	1.231	(0.992, 1.528)	915	0.001	1.382	(1.143, 1.671)	260	0.560	0.889	(0.597, 1.322)
	25–34	984	0.889	1.016	(0.808, 1.278)	1239	0.000	1.660	(1.370, 2.012)	354	0.101	1.378	(0.940, 2.022)
	35–44	1019	0.439	1.106	(0.857, 1.427)	1748	0.001	1.439	(1.166, 1.776)	426	0.128	1.393	(0.909, 2.136)
	45–54	1031	0.325	1.121	(0.893, 1.409)	1837	0.001	1.361	(1.134, 1.633)	472	0.039	1.475	(1.020, 2.134)
	55–64	713	0.295	1.140	(0.892, 1.455)	1614	0.101	1.170	(0.970, 1.411)	402	0.932	1.017	(0.690, 1.500)
Parent-hood	Parents	1833	0.039	1.189	(1.008, 1.401)	2871	0.328	1.077	(0.928, 1.251)	726	0.637	0.934	(0.705, 1.238)
faith Education			0.000				0.000				0.287		
	Ultra-orthodox	190	0.004	0.613	(0.441, 0.852)	471	0.046	0.786	(0.621, 0.996)	110	0.291	1.269	(0.816, 1.973)
	Religious	515	0.012	0.769	(0.625, 0.944)	946	0.502	0.942	(0.793, 1.120)	235	0.773	0.951	(0.676, 1.337)
	Traditional	1349	0.000	1.323	(1.154, 1.516)	2373	0.000	1.275	(1.132, 1.436)	569	0.107	1.215	(0.959, 1.539)
			0.116				0.000				0.007		
	Non Academic	1582	0.993	1.001	(0.874, 1.001)	4862	0.838	0.972	(0.743, 1.272)	1277	0.774	1.079	(0.642, 1.813)
Income	Academic	1179	0.051	0.856	(0.795, 1.057)	3658	0.024	0.728	(0.552, 0.959)	827	0.288	0.747	(0.437, 1.279)
			0.000				0.000				0.003		
	Low	930	0.003	1.301	(1.091, 1.551)	1714	0.000	1.415	(1.219, 1.641)	411	0.002	1.590	(1.185, 2.133)
	Medium-High	2413	0.231	0.917	(0.795, 1.057)	3805	0.830	1.014	(0.891, 1.154)	954	0.844	1.026	(0.795, 1.324)
	High	425	0.001	0.652	(0.507, 0.837)	907	0.050	0.814	(0.662, 1.000)	217	0.527	0.876	(0.580, 1.322)
constant			0.000	0.336			0.000	0.156			0.000	0.174	

Note: Abbreviations: *p*, *p*-value; OR, Odds Ratio; CI, 95% Confidence Intervals; SES, Socioeconomic status. “During LICs” Includes the Second Lebanon War.

**Table 6:** Binary logistic model predicting “fear of the future” during LICs and during routine, stratified analysis.

Covariate	Levels of Covariate	During Routine (N = 8400)				During LICs (N = 11,907)				During the Second Lebanon War (N = 4001)			
		*n*	*p*	OR	95% CI	*n*	*p*	OR	95% CI	*n*	*p*	OR	95% CI
Gender	Female	4939	0.000	1.830	(1.679, 1.994)	7551	0.000	2.607	(2.417, 2.813)	2451	0.000	2.958	(2.597, 3.370)
Age	Age		0.101				0.004				0.012		
	18–24	1330	0.584	0.961	(0.835, 1.107)	1289	0.271	1.079	(0.942, 1.236)	473	0.903	1.016	(0.792, 1.302)
	25–34	1332	0.982	0.998	(0.856, 1.164)	1733	0.001	1.260	(1.096, 1.449)	632	0.165	1.197	(0.929, 1.542)
	35–44	1893	0.064	1.157	(0.992, 1.350)	2346	0.001	1.310	(1.123, 1.528)	799	0.003	1.517	(1.147, 2.006)
	45–54	1504	0.366	1.075	(0.919, 1.259)	2491	0.000	1.270	(1.113, 1.450)	873	0.009	1.385	(1.086, 1.766)
	55–64	989	0.211	1.119	(0.938, 1.335)	2126	0.049	1.145	(1.001, 1.331)	731	0.084	1.244	(0.971, 1.594)
Parent-hood	Parents	2380	0.710	1.022	(0.910, 1.148)	3917	0.018	1.143	(1.023, 1.277)	1359	0.078	1.183	(0.981, 1.426)
faith Education			0.000				0.000				0.062		
	Ultra-orthodox	401	0.000	0.682	(0.556, 0.837)	616	0.000	0.649	(0.544, 0.774)	187	0.484	0.894	(0.652, 1.224)
	Religious	1007	0.000	0.743	(0.649, 0.850)	1268	0.011	0.850	(0.750, 0.964)	437	0.968	0.996	(0.804, 1.234)
	Traditional	2138	0.004	1.166	(1.051, 1.294)	3205	0.022	1.110	(1.015, 1.214)	1071	0.016	1.213	(1.037, 1.420)
			0.500				0.000				0.041		
	Non Academic	3426	0.249	0.940	(0.847, 1.044)	6248	0.001	0.804	(0.706, 0.915)	2397	0.436	0.859	(0.585, 1.260)
Income	Academic	2465	0.411	0.953	(0.850, 1.069)	4521	0.000	0.721	(0.630, 0.826)	1471	0.106	0.723	(0.488, 1.071)
			0.242				0.000				0.002		
	Low	1666	0.183	0.915	(0.802, 1.043)	2379	0.000	1.295	(1.157, 1.449)	799	0.001	1.426	(1.166, 1.745)
	Medium-High	3848	0.043	0.894	(0.801, 0.996)	5191	0.370	1.044	(0.950, 1.146)	1797	0.378	1.079	(0.911, 1.277)
	High	823	0.320	0.920	(0.781, 1.084)	1268	0.484	0.951	(0.826, 1.094)	443	0.739	0.959	(0.751, 1.225)
constant			0.157	0.899			0.000	0.523			0.000	0.471	

Note: Abbreviations: *p*, *p*-value; OR, Odds Ratio; CI, 95% Confidence Intervals; SES, Socioeconomic status. “During LICs” Includes the Second Lebanon War.

## Data Availability

Not applicable.

## References

[1] U.S. Army (1990). Military Operations in Low Intensity Conflict.

[2] Gray C.S. (2001). Thinking asymmetrically in times of terror. Parameters U.S. Army War Coll..

[3] Hoffman F.G. (2007). Conflict in the 21st Century: The Rise of Hybrid Wars.

[4] Michael K., Kellen D., Ben Ari E., Michael K., Kellen D., Ben Ari E. (2009). Introduction: Wars and peace support operations in the contemporary wars: Conceptual clarifications and suggestions. The Transformation of the World of War and Peace Support Operations.

[5] Smith R. (2007). The Utility of Force: The Art of War in the Modern World.

[6] Tiargan-Orr R., Eran-Jona M. (2016). The Israeli public’s perception of the IDF: Stability and change. Armed Forces Soc..

[7] Bar-Tal D. (2004). The necessity of observing real life situations: Palestinian-Israeli violence as a laboratory for learning about social behavior. Eur. J. Soc. Psychol..

[8] Sharvit K., Bar-Tal D., Raviv A., Raviv A., Gurevich R. (2010). Ideological orientation and social context as moderators of the effect of terrorism: The case of Israeli-Jewish public opinion regarding peace. Eur. J. Soc. Psychol..

[9] Eran-Jona M. (2011). Married to the military: Military-family relations in the Israel defense forces. Armed Forces Soc..

[10] Lavee Y., Ben David A. (2006). Families under war: Stresses and strains, Israeli families during the Gulf war. J. Trauma. Stress.

[11] Shoshani A., Slone M. (2016). The resilience functions of character strengths in the face of war and protracted conflict. Front. Psychol..

[12] Bleich A., Gelkopf M., Solomon Z. (2003). Exposure to terrorism, stress-related mental health symptoms, and coping behaviors among a nationally representative sample in Israel. J. Am. Med. Assoc..

[13] Bleich A., Gelkopf M., Melamed Y., Solomon Z. (2006). Mental health and resiliency following 44 months of terrorism: A survey of an Israeli national representative sample. BMC Med..

[14] Goral A., Feder-Bubis P., Lahad M., Galea S., O’Rourke N., Aharonson-Daniel L. (2021). Development and validation of the Continuous Traumatic Stress Response scale (CTSR) among adults exposed to ongoing security threats. PLoS ONE.

[15] Bonanno G.A. (2004). Loss, trauma, and human resilience: Have we underestimated the human capacity to thrive after extremely aversive events?. Am. Psychol..

[16] Bonanno G.A. (2005). Resilience in the face of potential trauma. Curr. Dir. Psychol. Sci..

[17] Bonanno G.A., Galea S., Bucciarelli A., Vlahov D. (2006). Psychological resilience after disaster: New York City in the after math of the September 11th terrorist attack. Psychol. Sci..

[18] Canetti D., Waismel-Manor I., Cohen N., Rapaport C. (2014). What does national resilience mean in a democracy? Evidence from the United States and Israel. Armed Forces Soc..

[19] Halperin E., Sharvit K. (2015). The Social Psychology of Intractable Conflicts.

[20] Halperin E. (2016). Emotions in Conflict: Inhibitors and Facilitators of Peace Making.

[21] Fredrickson B.L., Tugade M.M., Waugh C.E., Larkin G.R. (2003). What good are positive emotions in crisis? A prospective study of resilience and emotions following the terrorist attacks on the United States on September 11th, 2001. J. Personal. Soc. Psychol..

[22] Galea S., Ahern J., Resnick H., Kilpatrick D., Bucuvalas M., Gold J., Vlahov D. (2002). Psychological sequel of the September 11 terrorist attacks in New York City. N. Engl. J. Med..

[23] Gelkopf M., Solomon Z., Bleich A. (2013). A longitudinal study of changes in psychological responses to continuous terrorism. Isr. J. Psychiatry Relat. Sci..

[24] Krippner S., Mcintyre T. (2003). The Psychological Impact of War Trauma on Civilians: An International Perspective.

[25] Schuster M.A., Stein B.D., Jaycox L., Collins R.L., Marshall G.N., Elliott M.N., Zhou A.J., Kanouse D.E., Morrison J.L., Berry S.H. (2001). A national survey of stress reactions after the September 11, 2001, terrorist attacks. N. Engl. J. Med..

[26] Silver R.C., Holman E.A., McIntosh D.N., Poulin M., Gil-Rivas V. (2002). Nationwide longitudinal study of psychological responses to September11. J. Am. Med. Assoc..

[27] Sinclair S.J., Antonius D. (2012). The Psychology of Terrorism Fears.

[28] Bar-Tal D. (2001). Why does fear override hope in societies engulfed by intractable conflict, as it does in the Israeli society?. Political Psychol..

[29] Cohen M., Yahav R. (2008). Acute stress symptoms during the second Lebanon war in a random sample of Israeli citizens. J. Trauma. Stress.

[30] García-Ponce O., Young L., Zeitzoff T. (2018). Anger and Support for Punitive Justice in Mexico’s Drug War.

[31] Hobfoll S.E., Palmieri P.A., Johnson R.J., Canetti-Nisim D., Hall B.J., Galea S. (2009). Trajectories of resilience, resistance, and distress during ongoing terrorism: The case of Jews and Arabs in Israel. J. Consult. Clin. Psychol..

[32] Kaplan Z., Matar M.A., Kamin R., Sadan T., Cohen H. (2005). Stress-related responses after three years of exposure to terror in Israel: Are ideological-religious factors associated with resilience. J. Clin. Psychiatry.

[33] Lomranz J., Hobfoll S., Johnson R., Eyal N., Zemach M. (1994). A nation’s response to attack: Israelis’ depressive reactions to the gulf war. J. Trauma. Stress.

[34] Mandel R. (1991). Public Opinion and Superpower Strategic Amns. Armed Forces Soc..

[35] Sakstrup C., Hansen K.J. (2021). Revisiting the Emotion–Risk Interaction: Do Anger and Fear Moderate the Impact of Risk on Public Support for War?. Int. J. Public Opin. Res..

[36] Shalev A.Y., Freedman S. (2005). PTSD following terrorist attacks: A prospective evaluation. Am. J. Psychiatry.

[37] Benight C.C., Freyaldenhoven R.W., Hughes J., Ruiz J.M., Zoschke T.A. (2000). Coping self-efficacy and psychological distress following the Oklahoma City bombing. J. Appl. Soc. Psychol..

[38] Hobfoll S.E. (1998). Stress, Culture and Community: The Psychology and Philosophy of Stress.

[39] Hobfoll S.E., Canetti-Nisim D., Johnson R.J. (2006). Exposure to terrorism, stress-related mental health symptoms, and defensive coping among Jews and Arabs in Israel. J. Consult. Clin. Psychol..

[40] Hobfoll S.E. (1989). Conservation of resources: A new attempt at conceptualizing stress. Am. Psychol..

[41] Hobfoll S.E. (2001). The influence of culture, community, and the nested-self in the stress process: Advancing conservation of resources theory. Appl. Psychol..

[42] Granot H. (2011). The Golden Hour Individual & Community in Emergencies.

[43] Hobfoll S.E., Tracy M., Galea S. (2006). The impact of resource loss and traumatic growth on probable PTSD and depression following terrorist attacks. J. Trauma Stress.

[44] Shamai M., Kimhi S. (2007). Teenagers’ response to threat of war and terror: Gender and the role of social systems. Community Ment. Health J..

[45] Hobfoll S.E. (1986). Stress, Social Support, and Women.

[46] Maguire B., Hagan P. (2007). Disasters and communities: Understanding social resilience. Aust. J. Emerg. Manag..

[47] Solomon Z., Gelkopf M., Bleich A. (2005). Is terror gender-blind? Gender differences in reaction to terror events. Soc. Psychiatry Epidemiol..

[48] Slone M., Shoshani A. (2014). Psychiatric effects of protracted conflict and political life events exposure among adolescents in Israel: 1998–2011. J. Trauma. Stress.

[49] Lahad M., Leykin D. (2010). Ongoing exposure versus intense periodic exposure to military conflict and terror attacks in Israel. J. Trauma. Stress.

[50] Scott T. (2010). Religion in trauma care: Grand narratives and sacred rituals. Trauma.

[51] Kovatz S., Kutz I., Rubin G., Dekel R., Shenkman L. (2006). Comparing the distress of American and Israeli medical students studying in Israel during a period of terror. Med. Educ..

[52] Jarymowicz M., Bar-Tal D. (2006). The dominance of fear over hope in the life of individuals and collectives. Eur. J. Soc. Psychol..

[53] Pliskin R., Sheppes G., Halperin E. (2015). Running for your life, in context: Are rightists always less likely to consider fleeing their country when fearing future events?. J. Exp. Soc. Psychol..

[54] Mueller J.E. (1970). Presidential Popularity from Truman to Johnson. Am. Political Sci. Rev..

[55] Mueller J.E. (1973). War, President and Public Opinion.

[56] Hobfoll S.E., Lomranz J., Eyal N., Bridges A., Tzemach M. (1989). Pulse of a nation: Depressive mood reactions of Israelis to the Israel-Lebanon War. J. Personal. Soc. Psychol..

